# Bioinformatics Prediction and Machine Learning on Gene Expression Data Identifies Novel Gene Candidates in Gastric Cancer

**DOI:** 10.3390/genes13122233

**Published:** 2022-11-28

**Authors:** Medi Kori, Esra Gov

**Affiliations:** 1 Department of Bioengineering, Marmara University, Istanbul 34854, Turkey; 2 Department of Bioengineering, Adana Alparslan Türkeş Science and Technology University, Adana 01250, Turkey

**Keywords:** gastric cancer, disease genes, diagnostic genes, prognostic genes, multi-omics, systems biology

## Abstract

Gastric cancer (GC) is one of the five most common cancers in the world and unfortunately has a high mortality rate. To date, the pathogenesis and disease genes of GC are unclear, so the need for new diagnostic and prognostic strategies for GC is undeniable. Despite particular findings in this regard, a holistic approach encompassing molecular data from different biological levels for GC has been lacking. To translate Big Data into system-level biomarkers, in this study, we integrated three different GC gene expression data with three different biological networks for the first time and captured biologically significant (i.e., reporter) transcripts, hub proteins, transcription factors, and receptor molecules of GC. We analyzed the revealed biomolecules with independent RNA-seq data for their diagnostic and prognostic capabilities. While this holistic approach uncovered biomolecules already associated with GC, it also revealed novel system biomarker candidates for GC. Classification performances of novel candidate biomarkers with machine learning approaches were investigated. With this study, AES, CEBPZ, GRK6, HPGDS, SKIL, and SP3 were identified for the first time as diagnostic and/or prognostic biomarker candidates for GC. Consequently, we have provided valuable data for further experimental and clinical efforts that may be useful for the diagnosis and/or prognosis of GC.

## 1. Introduction

Gastric cancer (GC) is one of the leading causes of cancer deaths worldwide with a high prevalence. According to recent reports, GC is responsible for one in 13 deaths worldwide and was the fifth most common cancer worldwide in 2020 [1]. Helicobacter pylori infection is the main risk factor for GC, but other factors such as genetic and environmental factors also play a role [2]. Because GC is a heterogeneous disease, it is an attractive model for studying carcinogenesis and tumorigenesis. The exact mechanisms underlying the development of GC are still unknown despite significant progress in understanding the molecular causes of GC. Malignant transformation of gastric mucosa during the multistep process of GC pathogenesis is caused by a variety of genetic and molecular abnormalities that occur in GC [3]. Since one of the major causes of treatment failure in GC is drug resistance, a deeper knowledge of novel gene candidates is crucial to better understand the molecular mechanism of pathogenesis, which could improve patient survival [4].

Integrating multi-omics data can reveal the entire physical and functional architecture of cellular signaling and regulatory pathways. Moreover, it has been reported that a systems medicine approach involving the integration of gene expression data with multi-omics data reveals important and crucial genes in a pathological state [5]. Today, systems medicine is being used in several studies to identify significant disease genes, for example, in papillary thyroid cancer [6], acute myeloid leukemia [7], abdominal aortic aneurysm [8], ovarian cancer stem cells [9], rheumatoid arthritis [10], colorectal cancer [11], and three different ovarian diseases [12].

The rapid development of cancer genomics has led to extensive information on crucial genes in malignancies. Although many bioinformatics web servers and tools have been developed to identify disease genes [13,14], there are still no clinically validated treatments and their efficacy is controversial for the vast majority of cancer genes. Recently, key genes [15], prognostic genes [16], and prognostic and diagnostic genes for GC [17] have been reported. In another study conducted by our research group, we analyzed several microarray datasets of GC and identified a prognostic differential co-expressed gene module and offered drug candidates by repurposing analysis [18].

**Figure 1:**
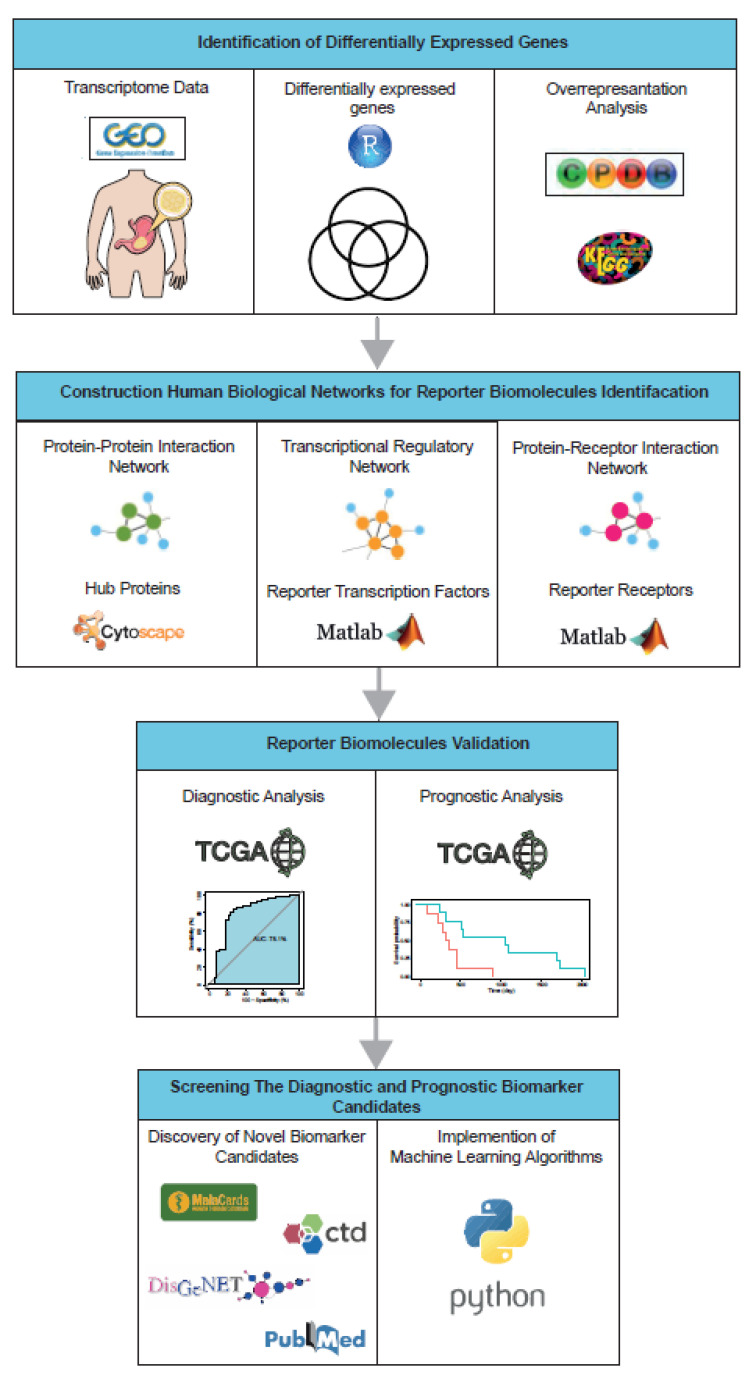
The computational flow employed in the study.

Although several studies have been performed to identify disease genes in GC, the pathogenesis of GC is still unclear and new and efficient biomarker candidates are needed. In parallel with the advances in high-throughput technologies and the increasing number of omics data, the increasing use of systems biology approaches to understand diseases at the systems level and provide biomarker candidates is becoming increasingly important. In our study, we used an integrative multi-omics approach that differs from previous GC studies. In the present study, we adopted a systems biology approach by integrating gene expression data with comprehensive human biological networks to identify molecular signatures that allow us to identify important biomolecules which can be considered as biomarkers associated with GC. Accordingly, a meta-analysis of GC-associated transcriptomic datasets was performed and common differentially expressed genes (DEGs) were identified among the datasets. Gene set overrepresentation analysis was performed for the common DEGs. The DEGs were integrated into various human biological networks, including protein–protein interactions (PPI), transcriptional regulation, and protein–receptor interaction networks to identify hub proteins, reporter transcription factors (TFs), and reporter receptors. The diagnostic and prognostic value of reporter biomolecules was assessed using an independent cohort study. Finally, the novel prognostic and diagnostic biomarker candidates for GC were revealed and their classification power was evaluated using machine learning approaches (Figure 1). Consequently, we believe that the novel biomarker candidates presented here will be a crucial resource for understanding the pathogenesis of GC and can be considered as powerful diagnostic and prognostic biomarkers for further experimental and clinical studies for GC.

## 2. Materials and Methods

### 2.1. Gene Expression Datasets of Gastric Carcinoma

Three microarray datasets, including GSE19826 [19], GSE54129 (unpublished), and GSE79973 [20] from independent studies, were obtained from the Gene Expression Omnibus (NCBI-GEO) database [21] to meta-analyze transcriptome profiles in GC. Datasets were selected based on the following criteria: (i) samples consisted of two different phenotypes (i.e., cancerous vs. normal); (ii) each phenotype included at least 3 samples; (iii) the microarray platforms used were from the same platform. A total of 179 samples were analyzed, including 133 GC and 46 normal gastric tissue samples.

An independent adenocarcinoma dataset of the stomach (STAD) from The Cancer Genome Atlas (TCGA) [22], comprising 375 GC and 32 normal stomach tissue samples, was used as a validation dataset, for preclinical validation purposes (i.e., diagnostic and prognostic analyses), and for implementing machine learning approaches.

### 2.2. Identification of Differentially Expressed Genes

In this study, a well-established statistical analysis procedure [23,24] was used to identify DEGs. Briefly, the raw data (stored in CEL files) of each dataset were normalized by calculating the Robust Multi-Array Average (RMA) expression measure [25] implemented in the Affy package [26] of the R/Bioconductor platform (version 4.0.2) [27]. DEGs were identified from normalized expression values using the Linear Models for Microarray Data package (LIMMA) [28]. The Benjamini–Hochberg method was used to control for the false discovery rate (FDR). The adjusted *p*-value < 0.05 was used as the cutoff value to determine the statistical significance of the DEGs. To determine the regulatory patterns of DEGs, the fold-change thresholds were used as 2-fold change. Each data set was analyzed independently, and the results were comparatively analyzed to identify common signatures from these independent studies, and common DEGs were used in further analyses.

### 2.3. Gene Set Overrepresentation Analyses

Overrepresentation analyses were performed using ConsensusPathDB [29] to determine the functional annotations (i.e., biological pathways) of the DEGs. In the analyses, KEGG [30] and Reactome [31] were employed as the data sources for pathways The *p*-values were determined using Fisher’s exact test, and a false discovery rate was applied to control the *p*-values. An adjusted *p*-value < 0.01 was considered statistically significant.

### 2.4. Reconstruction of Protein–Protein Interaction Network and Identification of Hub Proteins

Physical PPIs among DEGs were extracted from the BioGRID database (MV-Physical-4.2.191) [32], which contains 51,745 physical and experimentally detected PPIs among 10,177 human proteins. The PPI sub-network was reconstructed for common DEGs with their first neighbors and visualized using Cytoscape (v3.5.0) [33]. To determine hub proteins (i.e., central proteins), topological analyses were performed using the Cytohubba plugin [34]. The dual metric approach that considers degree and betweenness centrality metrics (i.e., degree as a local metric and betweenness centrality as a global metric) was simultaneously used to identify hub proteins. The 10 proteins with the highest degree and betweenness centrality values in the PPI subnetwork were determined as hub proteins.

### 2.5. Identification of Reporter Transcription Factors and Receptors

The reporter molecules were identified using the reporter features algorithm [35], which was previously adapted for potential TFs and receptors [36]. Briefly, reporters were identified by integrating common DEGs gene expression data with relevant human biological networks (i.e., TF-target gene interactions and receptor–protein interactions). TF–target gene interaction information was obtained from the TRRUST (transcriptional regulatory relationships unraveled by sentence-based text-mining) database [37]. The proteins with receptor activity (GO: 0004872) were extracted from DAVID [38], PANTHER [39], and GeneCodis [40] databases, and the physical interactions of these receptors were extracted from the human PPI network [32]. The reporter features algorithm [35] was implemented in MATLAB (R2016). The *p*-values of the calculated reporter molecules were controlled with FDR, and the reporter molecules with adjusted *p*-value < 0.001 were considered significant. Reporter TFs and receptors functions were analyzed using the PANTHER classification system [39].

### 2.6. Pre-Clinical Diagnostic Validation of Reporter Biomolecules

To evaluate the diagnostic performance of reporter biomolecules (i.e., hub proteins, TFs, and receptors), a receiver-operating characteristic curve (ROC) was used that utilized the parameters of sensitivity and specificity to predict diagnostic ability. To determine the overall diagnostic accuracy of diagnostic performance, the area under the roc curve (AUC) was calculated. A reporter biomolecule with an AUC value ≥ 70% was considered statistically significant [41] and accepted as a diagnostic biomolecule.

### 2.7. Pre-Clinical Prognostic Validation of Reporter Biomolecules

To evaluate the prognostic performance of the reporter biomolecules (i.e., hub proteins, TFs, and receptors), we obtained clinical information from STAD samples of TCGA [22] and used it in the prognostic performance analyses. To determine the prognostic performance of each biomolecule, survival analyses were performed by dividing subjects into two groups (high and low risk) according to their prognostic index (PI), which is the linear component of the Cox model. The differences in gene expression values between the risk groups were represented by box plots. Survival signatures of reporter biomolecules were evaluated by Kaplan–Meier plots. The hazard ratio (HR = (O1/E1)/(O2/E2)) was calculated using the ratio between the relative mortality rate in group 1 and the relative mortality rate in group 2, where O and E are the observed and expected number of deaths, respectively. Reporter biomolecules with a log-rank *p*-value < 0.05 were considered statistically significant and accepted as prognostic biomolecules.

### 2.8. Screening the Association of Diagnostic and Prognostic Reporter Biomolecules with Gastric Cancer

Following the two preclinical validation analyses, an extensive search was performed to determine whether the diagnostic or/and prognostic reporter biomolecules found in the study had been previously associated with GC. The following databases and electronic search services were used throughout the association screening process: Malacards: The Human Disease Database [42], DisGeNET: a comprehensive platform for integrating information on genes and variants associated with human diseases [43], Comparative Toxicogenomics Database (CTD) [44], PubMed, Science-Direct, Scopus, and Web of Science. The reporter biomolecules, which have diagnostic or/and prognostic capabilities and were not associated with GC according to previous studies, were considered as novel biomarker candidates in this study.

### 2.9. Investigation of Classification Performances of Novel Candidate Biomarkers with Machine Learning Approaches

To better interpret new biomarker candidates, we applied several classification methods, a well-known and useful machine learning technique in biomarker discovery, to identify novel biomarker candidates. We implemented different classification algorithms, including K-Neighbors, MLP, Decision Tree, Random Forest, Gradient Boosting, CatBoost, LGBM, and XGB using the Python programming language [45]. The performance of these techniques was estimated based on the predictive accuracy of the classifiers.

## 3. Results

### 3.1. The Transcriptomic Signatures of Gastric Cancer: Identification of Differentially Expressed Genes

**Figure 2:**
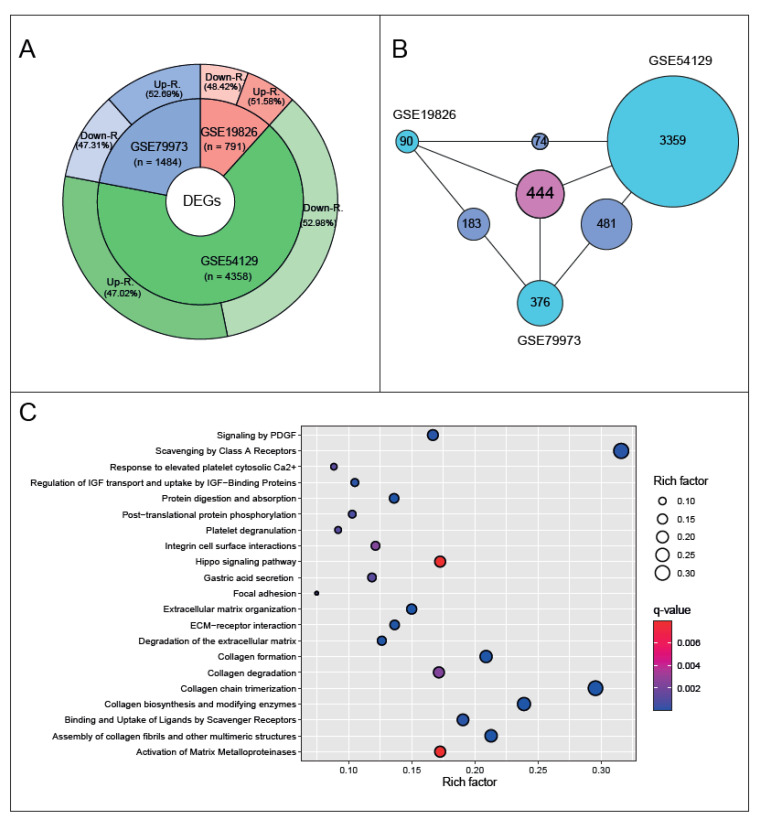
Meta-analysis of the three transcriptome datasets associated with gastric cancer. (**A**) Pie donut diagram shows the distribution of differentially expressed genes (DEGs) of the three transcriptome datasets. (**B**) The Venn diagram shows the DEGs common to the datasets. (**C**) The gene set overrepresentation analysis of the common DEGs.

The individual statistical analyses of three gene expression datasets (GSE19826, GSE54129, and GSE79973) led to the identification of DEGs. The number of DEGs in each dataset showed a wide range from 791 to 4358 genes, and the highest number of DEGs was identified in GSE54129. In all three datasets, no significant tendency toward a particular regulatory pattern (up- or down-regulation) was detected in the culminated DEGs; in other words, the difference between up- and down-regulation did not exceed 5%. Nevertheless, in both datasets (i.e., GSE19826 and GSE79973), up-regulated DEGs predominated (51.4% and 52.6%, respectively) compared with down-regulated DEGs. On the other hand, DEGs in dataset GSE54129 showed a stronger pattern of down-regulation compared with up-regulation (Figure 2A).

Excluding the regulatory patterns of DEGs, the comparative analysis of the resulting DEGs showed that a total of 444 DEGs were common in the three GEO datasets (Figure 2B). To ensure consistency of the analysis, further analyses were performed using these common DEGs.

Overrepresentation analyses indicated that the common DEGs were significantly associated with cancer-associated molecular pathways, such as Hippo signaling, focal adhesion extracellular matrix (ECM) receptor interaction. Several processes that were associated with collagen synthesis or degradation were highlighted as pathways for common DEGs. Moreover, gastric acid segregation and protein digestion and the absorption pathway, which were highly associated with each other, come into prominence in overrepresentation analysis (Figure 2C).

### 3.2. The Proteomic Signatures of Gastric Cancer: Identification of Hub Proteins

To identify hub proteins, a PPI subnetwork was reconstructed around proteins encoded by the common DEGs of GC. The reconstructed network consisted of 974 proteins (i.e., 444 common proteins and their physically interacting first neighbors) and 1025 links (i.e., physical PPIs between these proteins).

**Figure 3:**
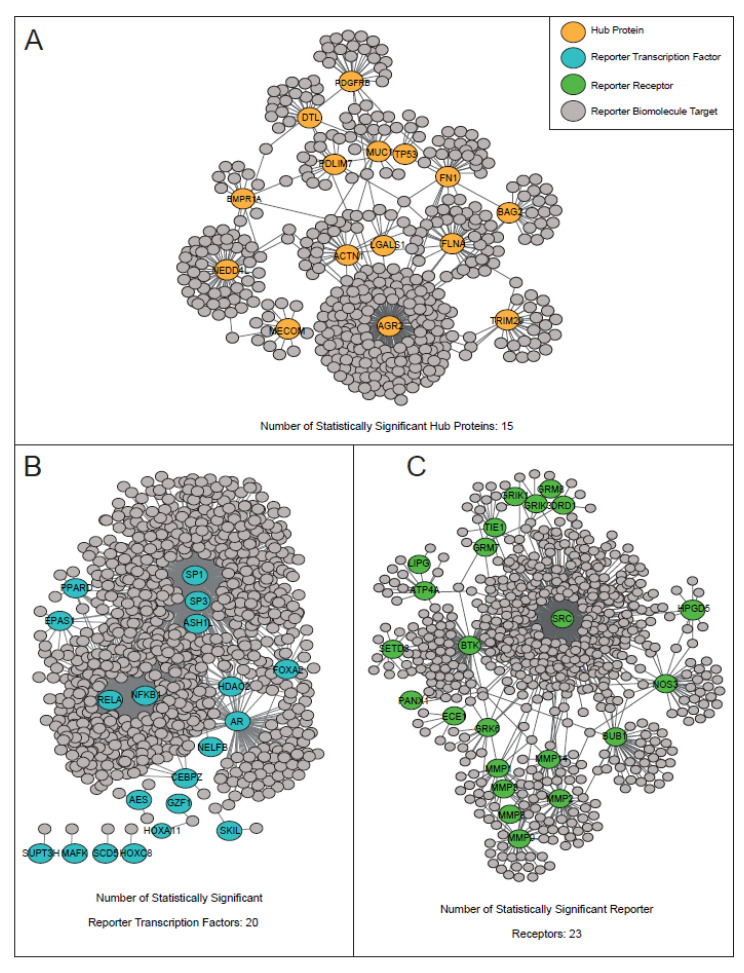
The reconstructed human biological networks. (**A**) The reconstructed protein–protein interaction (PPI) network. The revealed significant hub proteins according to employed topological parameters were shown in orange. (**B**) The reconstructed transcriptional regulatory interaction network. The statistically significant (*p*-value < 0.001) reporter transcription factors (TFs) were shown in blue. (**C**) The reconstructed protein–receptor interaction network interaction network. The statistically significant (*p*-value < 0.001) reporter receptors were shown in green.

The PPI network showed a scale-free topology and indicated the presence of hub proteins. Hub proteins, which play a central role in modular organization and information flow within the network, were identified by topological analysis that included degree and betweenness centrality metrics. The 10 proteins with the highest degree and betweenness centrality values were combined together and determined as hub proteins. As a result, a total of 15 hub proteins were determined, namely ACTN1, AGR2, BAG2, BMPR1A, DTL, FLNA, FN1, LGALS1, MECOM, MUC1, NEDD4L, PDGFRB, PDLIM7, TP53, and TRIM29 (Figure 3A).

### 3.3. The Regulatory Signatures of Gastric Cancer: Identification of Reporter Transcription Factors

The regulatory elements (i.e., TFs) controlling key transcriptional changes in GC genes were identified by integrating common DEGs with the transcriptional regulatory network using the reporter features algorithm. Accordingly, 20 TFs emerged with a significance level of *p*-value < 0.001 and were identified as reporter regulatory elements in the transcriptional control of genes in GC (Figure 3B). These reporter TFs included two rel homology transcription factors (NFKB1 and RELA), two zinc finger transcription factors (SP1 and SP3), and two DNA-binding transcription factors (CEBPZ and GZF1).

### 3.4. The Signaling Signatures of Gastric Cancer: Identification of Reporter Receptors

Reporter receptors of GC were determined in a similar manner that was used to determine reporter TFs. To identify reporter receptors, we integrated common DEGs with the receptor–protein interaction network by using the reporter features algorithm. According to the results, 23 proteins were identified as reporter receptors with a significance level of *p*-value < 0.001 (Figure 3C). Among the 23 reporter receptors, seven proteins belonged to the metalloprotease family (ECE1, MMP1, MMP14, MMP2, MMP3, MMP8, and MMP9), three reporter receptors belonged to the transmembrane signaling receptors (GRIK1, GRIK3, and TIE1), and three reporter receptors belonged to the G protein-coupled receptors (DRD1, GRM7, and GRM8).

### 3.5. Diagnostic and Prognostic Power of Reporter Biomolecules of Gastric Cancer

**Figure 4:**
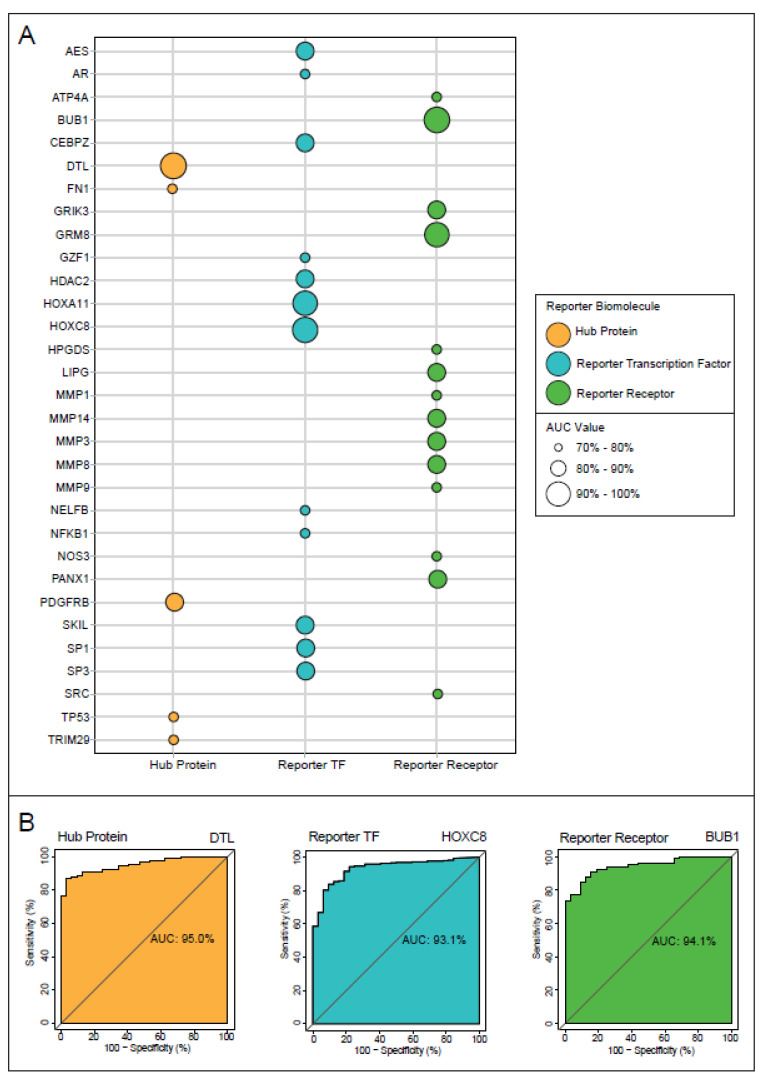
The diagnostic performance analyses of the reporter biomolecules. (**A**) The bubble plot representing the AUC values of the reporter biomolecules. Only the AUC values that were considered significant in the study were shown (AUC > 70%). Hub proteins are shown in orange, reporter transcription factors (TFs) in blue, and reporter receptors in green. (**B**) The major reporter biomolecules: a hub protein, a TF, and a reporter receptor according to their AUC values.

To pre-clinically validate the diagnostic and prognostic capabilities of the discovered reporter biomolecules, independent expression data from TCGA (TCGA-STAD) were used. The diagnostic property of each module was evaluated using ROC curves, and a reporter biomolecule with an AUC value ≥ 70% was considered statistically significant and accepted as diagnostic. Subsequently, five hub proteins (33.3% of total hubs), 12 reporter TFs (60% of total reporter TFs), and 14 reporter receptors (60.8% of total reporter receptors) were found to be diagnostic reporter biomolecules (Figure 4A). Among the diagnostic biomolecules, a hub protein, DTL, with an AUC score of 95%, a reporter TF, HOXC8, with an AUC score of 93.1%, and a reporter receptor, BUB1, with an AUC score of 94.1% were the most important diagnostic reporter biomolecules when statistical significance was considered (Figure 4B).

**Figure 5:**
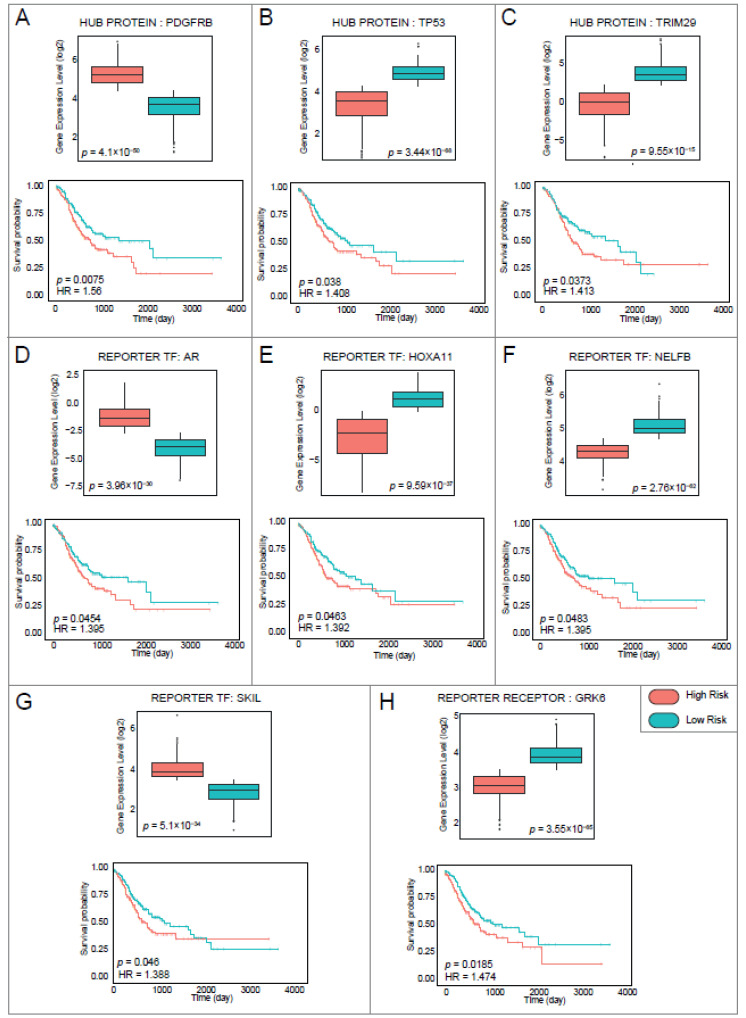
Analysis of prognostic performance of reporter biomolecules. Box plots showing expression levels of reporter biomolecules between low and high-risk groups with *p*-values. Kaplan–Meier plots estimating survival of patients with gastric cancer showing *p*-value and hazard ratio for each curve. (**A**) Hub protein: PDGFRB. (**B**) Hub protein: TP53. (**C**) Hub protein: TRIM29. (**D**) Reporter transcription factor (TF): AR. (**E**) Reporter TF: HOXA11. (**F**) Reporter TF: NELFB. (**G**) Reporter TF: SKIL. (**H**) Reporter receptor: GRK6. The high-risk group was shown in red, while the low-risk group was shown in blue.

Patient information on overall survival was extracted from data from TCGA-STAD and used for prognostic performance analysis. Prognostic performance of reporter biomolecules was assessed using Kaplan–Meier survival charts based on risk groups and days of survival. The log-rank *p*-value and hazard ratios were considered to determine whether reporter biomolecules had a high impact on overall patient survival. As a result, a total of three hub proteins (i.e., PDGFRB, TP53, and TRIM29), four reporter TFs (i.e., AR, HOXA11, NELFB, and SKIL), and one reporter receptor (GRK6) had a high impact on patients’ overall survival (log-rank *p*-value < 0.05). In addition, the differences in the expression levels of genes (encoding hub proteins, reporter TFs, or reporter receptors) between the risk groups showed that up-regulation of the expression of PDGFRB, AR, and SKIL was associated with a higher risk of GC, while down-regulation of the expression of TP53, TRIM29, HOXA11, NELFB, and GRK6 was associated with a higher risk of GC (Figure 5). With the exception of GRK6, all prognostic reporter biomolecules also showed high diagnostic performance. Thus, seven reporter biomolecules, namely AR, HOXA11, NELFB, PDGFRB, SKIL, TP53, and TRIM29 showed both statistically significant diagnostic and prognostic properties for GC. The reporter biomolecules that exhibited diagnostic or/and prognostic properties were considered as biomarker candidates for GC.

### 3.6. The Association of Diagnostic and Prognostic Reporter Biomolecules with Gastric Cancer

A total of 32 candidate biomarkers were identified by bioinformatics analysis. To determine whether the candidate biomarkers found were associated with GC from previous studies or were discovered for the first time with our study, we primarily examined GC-associated biomarkers and genes from three different publicly available databases. As a result, we obtained 1224 different GC-related biomarkers/genes from data repositories [42,43,44], including the 13 candidate biomarkers we proposed in this study. For the remaining 19 candidate biomarkers, we manually reviewed electronic search services and found that 13 candidate biomarkers had been previously associated with GC [46,47,48,49,50,51,52,53,54,55,56,57,58]. Consequently, we concluded that, to our knowledge, six diagnostic or/and prognostic reporter biomolecules, including AES, CEBPZ, GRK6, HPGDS, SKIL, and SP3, are proposed here for the first time as GC biomarker candidates (Table 1).

### 3.7. Classification Powers of Novel Candidate Biomarkers

**Figure 6:**
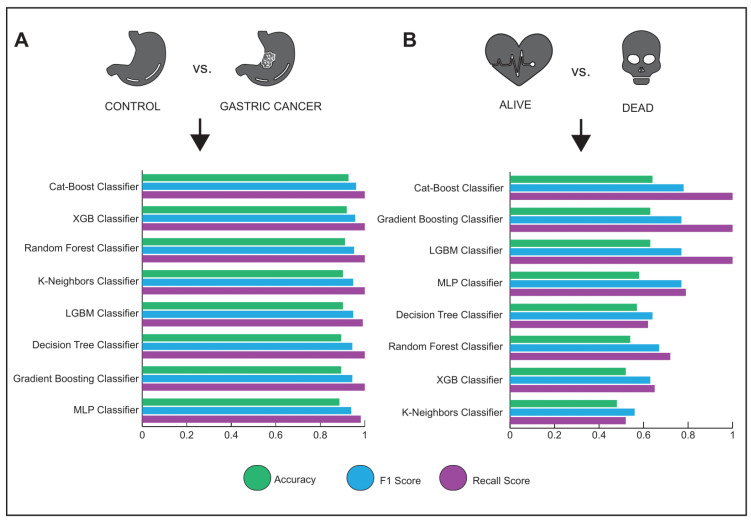
Machine learning analysis for novel diagnostic and/or prognostic biomarker candidates. (**A**) The accuracy, F1, and recall score plot of eight different classification algorithms for discriminating between diseased samples and controls. (**B**) The accuracy, F1, and recall score plot of eight different classification algorithms for discriminating between alive and dead samples.

A machine learning technique, classification, can be used to evaluate the potential of biomarkers identified by various statistical tests. Because an effective potential biomarker should be able to distinguish the diseased cohort from controls, we used several classification algorithms to determine the potential of our novel biomarkers. We evaluated the novel biomarker candidates based on the predictive accuracy of the classifier and found that the accuracy of the eight different classification methods ranged from 92.6% to 89.4% (Figure 6A), suggesting that the novel GC biomarkers we have provided here can efficiently discriminate the diseased samples from the controls. In addition, we used clinical data from our validation dataset (i.e., STAD-TCGA) [22] to test whether our candidates were informative in classifying alive and dead specimens. The accuracy of the classification results showed that our proposed novel biomarkers were not as successful in evaluating live and dead specimens compared with diseased and control specimens (accuracy ranged from 64.6% to 47.7%) (Figure 6B).

## 4. Discussion

It is estimated that GC will be responsible for 770,000 deaths and 1.1 million new cancer cases worldwide in 2020. Worse, it is predicted that by 2040, GC cases will result in approximately 1.3 million deaths and approximately 1.8 million people will be diagnosed with the disease [59]. Although many GC studies have accumulated in the scientific community to date, recent cancer statistics estimate the global burden of GC and clearly demonstrate the need for new diagnostic and prognostic strategies for GC. Despite these GC-based studies, the intertwined structure of the cell has not been considered, which requires the integration of biological data (i.e., expression data) with human biological networks. To translate Big Data into system-level biomarkers, in this study, we integrated GC expression data with three different biological networks for the first time and captured transcripts, hub proteins, TFs, and receptor molecules of GC. In addition, to determine a reporter biomolecule as a “biomarker,” we assessed its diagnostic and prognostic performance in an independent cohort.

Based on individual analysis of three gene expression datasets, we found that hundreds of genes were differentially expressed in each dataset. However, to increase the reliability and robustness of the results, we combine information from multiple microarray datasets and focus only on common 444 DEGs. Analysis of the overrepresentation of common DEGs revealed significant biological pathways. Interestingly, four pathways related to collagen synthesis or degradation were found to be significant. It was known that the restructuring of the collagen components of the tumor microenvironment had a remarkable impact on cancer development and progression. For GC, it was reported that the collagen components in the tumor microenvironment rearrange quantitatively and qualitatively, and there was a significant correlation between the prognosis of GC and collagen. The study even concluded that collagen width can be used as a prognostic indicator for GC [60].

Reconstruction and topological analysis of the PPI network around the proteins encoded by the 444 common DEGs led to the identification of hub proteins that play a central role in the flow of information within the network. A total of 15 hub proteins appeared as reporter signal mediators in GC. Among them, PDGFRB, TP53, and TRIM29 have shown both high diagnostic and prognostic capacity, while DTL and FN1 have shown only high diagnostic capacity. These diagnostic and/or prognostic biomarker candidates have already been associated with GC (Table 1), so these results further strengthen our confidence in our observations.

In this study, the reporter features algorithm was adapted to identify reporter TFs and receptors. Transcriptional expression of the common transcripts of GC was controlled by 20 TFs, whereas 23 receptors played a central role in signal transduction. Of these reporters, 12 TFs and 15 reporter receptors showed significant diagnostic and/or prognostic results when cross-validation analysis was performed with independent RNA-seq data. Accordingly, eight TFs and 13 reporter receptors have already been shown to be associated with GC (Table 1). However, to the best of our knowledge, AES, CEBPZ, GRK6, HPGDS, SKIL, and SP3 have not yet been associated with GC and were considered as novel biomarker candidates in this study. It was found that novel biomarker candidates efficiently discriminate the diseased samples from the controls compared to the performance of discriminating in the live and dead specimens. Since, it may suggest that biomarker candidates have the diagnostic capability of GC.

AES (also known as TLE5) is a transcriptional modulator and a transcriptional co-repressor that represses associated proteins of the Groucho/TLE family. AES plays an active role in the formation and development of organs or cells such as heart, pituitary, ear, and blood cells [61]. AES has been associated with several types of cancer. Deficiency of AES has been shown to lead to invasion and metastasis of prostate cancer [62]. Similarly, it suppresses colon cancer invasion and metastasis by inhibiting the Notch signaling pathway [63].

A TF, CEBPZ, acts as an activator or suppressor depending on the cell state, and its expression is associated with cellular stress, cell cycle arrest, or programmed cell death [64]. CEBPZ expression and methylation have been remarkably correlated with acute myeloid leukemia [65]. In a recent study, its overexpression was also found in squamous cell carcinoma of the esophagus [66].

GRK6 belongs to the family of G protein-coupled receptor kinases. It is overexpressed in immune cells and is closely associated with inflammation-related processes [67]. Up-regulation of GRK6 has been associated with colorectal cancer and is considered a potential biomarker for predicting poor survival in colorectal cancer patients [68]. In contrast, downregulation of GRK6 has been suggested as a potential biomarker for predicting overall survival in patients with lung adenocarcinoma [69]. In addition, up- or down-regulation of GRK6 expression has been observed in patients with hepatocellular carcinoma [70] and medulloblastoma [71], respectively.

HPGDS belongs to the family of transferases and catalyzes the production of a prostaglandin, prostaglandin D2, which is considered an essential lipid regulator that plays a remarkable role in the immune system, for example, in the inflammatory response [72]. According to a genome-wide association study, HPGDS was significantly associated with germ cell tumors in the testis [73]. Moreover, in a recent study, HPGDS was considered a prognostic biomarker for lung adenocarcinomas, and it was suggested that HPGDS may provide clues to the aggressiveness of the disease [74].

SKIL (also known as SnoN) is a transcriptional co-repressor that negatively regulates TGF-β signaling. SKIL has been associated with many cancers. For example, upregulation or amplification of SKIL has been associated with breast cancer [75], squamous cell carcinoma of the esophagus [76], prostate cancer, squamous cell carcinoma of the head and neck, and non-small cell lung cancer [77]. In addition, SKIL has been associated with leukemia [78], ovarian cancer [79], and squamous cell carcinoma of the lung [80].

SP3, which has a highly conserved DNA-binding domain, can either promote or repress the transcriptional activity of the corresponding target genes that play a role in the cell cycle, differentiation, or carcinogenesis [81]. High expression of SP3 was observed in hepatocellular carcinoma tissues compared with control tissues [82]. In another study, SP3 is described as a driving force for cancer metastasis in sarcomas [83].

In summary, we present here for the first time the molecular codes of GC at the different system levels (i.e., hub proteins, receptor TFs, and receptors) based on an integrative multi-omics approach and machine learning algorithms. The bioinformatics and machine learning approach determined previously identified biomolecules associated with GC as well as novel diagnostic and/or prognostic biomarker candidates such as AES, CEBPZ, GRK6, HPGDS, SKIL, and SP3. We believe that these results will provide insights into the underlying mechanisms of GC progression as well as some powerful novel biomarker candidates for GC. Despite the tremendous significance of the results of this study, further efforts are needed to experimentally and clinically validate the insights gained here.

## Figures and Tables

**Table 1:** The association of diagnostic and prognostic candidate biomarkers with gastric cancer.

Type	Name	Diagnostic?	Prognostic?	Association with Gastric Cancer
Hub Protein	DTL	+	−	[46]
	FN1	+	−	[42,43]
	PDGFRB	+	+	[42,43]
	TP53	+	+	[42,43,44]
	TRIM29	+	+	[47]
Reporter Transcription Factor	AES	+	−	Novel
	AR	+	+	[42,43]
	CEBPZ	+	−	Novel
	GZF1	+	−	[48]
	HDAC2	+	−	[49]
	HOXA11	+	+	[50]
	HOXC8	+	−	[51]
	NELFB	+	+	[52]
	NFKB1	+	−	[42,43]
	SKIL	+	+	Novel
	SP1	+	−	[53]
	SP3	+	−	Novel
Reporter Receptor	ATP4A	+	−	[42]
	BUB1	+	−	[42]
	GRIK3	+	−	[54]
	GRK6	−	+	Novel
	GRM8	+	−	[55]
	HPGDS	+	−	Novel
	LIPG	+	−	[56]
	MMP1	+	−	[42,43]
	MMP14	+	−	[42,43]
	MMP3	+	−	[42,43]
	MMP8	+	−	[57]
	MMP9	+	−	[42,43]
	NOS3	+	−	[42,43,44]
	PANX1	+	−	[58]
	SRC	+	−	[42,43]

## Data Availability

Publicly available datasets were analyzed in this study. The datasets analyzed during the current study are available in The Gene Expression Omnibus (https://www.ncbi.nlm.nih.gov/geo/, accessed on 14 April 2022) and The Genome Cancer Atlas (https://portal.gdc.cancer.gov/) (accessed on 23 May 2022). Protein interactome data are available in Biological General Repository for Interaction Datasets (https://thebiogrid.org, accessed on 25 July 2022).
